# Parasites of vectors - *Ixodiphagus hookeri *and its *Wolbachia *symbionts in ticks in the Netherlands

**DOI:** 10.1186/1756-3305-4-228

**Published:** 2011-12-07

**Authors:** Ellen Tijsse-Klasen, Marieta Braks, Ernst-Jan Scholte, Hein Sprong

**Affiliations:** 1 Laboratory for Zoonoses and Environmental Microbiology, National Institute for Public Health and Environment (RIVM), Bilthoven, The Netherlands; 2 National Centre for Monitoring of Vectors (CMV), New Food and Consumer Product Safety Authority (nVWA), Dutch Ministry of Economic Affairs, Agriculture and Innovation, Wageningen, the Netherlands

**Keywords:** Ixodiphagus hookeri, Ixodes ricinus, Parasitic wasp, Tick, *Wolbachia*

## Abstract

**Background:**

*Ixodiphagus hookeri *is a parasitic wasp of ixodid ticks around the world. It has been studied as a potential bio-control agent for several tick species. We suspected that the presence of *Wolbachia *infected *I. hookeri *eggs in ticks is responsible for incidental detection of *Wolbachia *DNA in tick samples.

**Methods:**

The 28S *rRNA *and 16S *rRNA *genes of a specimen of *I. hookeri *was amplified and sequenced. PCR on part of the 28S *rRNA *gene was used to detect parasitic wasp DNA in 349 questing *Ixodes ricinus *ticks from various sampling sites. Furthermore, the *wsp *gene of *Wolbachia *was sequenced from the *I. hookeri *specimen and a subset of ticks was tested using this marker.

**Results:**

Several sequences from tick specimens were identical to the *Wolbachia *sequence of the *I. hookeri *specimen. *Ixodiphagus hookeri *was detected in 9.5% of all tested ticks, varying between 4% and 26% depending on geographic location. Ten out of eleven sampling sites throughout the Netherlands were positive for *I. hookeri*. Eighty-seven percent of *I. hookeri-*positive but only 1.6% of *I. hookeri*-negative ticks were *Wolbachia *positive. Detection of *I. hookeri *DNA was strongly associated with the detection of *Wolbachia *in ticks.

**Conclusion:**

This is the first reported case of *I. hookeri *in the Netherlands. Furthermore *I. hookeri *harbours *Wolbachia *species and is broadly distributed in the Netherlands. While detection of *Wolbachia *DNA in ticks might often be due to parasitism with this wasp, other sources of *Wolbachia *DNA in ticks might exist as well.

## Background

### Ticks as vectors for disease

Ticks carry numerous microorganisms, ranging from highly pathogenic bacteria to intracellular symbionts. In addition to well-known pathogens including *Rickettsia *spp., *Borrelia burgdorferi *s.l. and *Anaplasma *spp., more and more tick-associated bacteria are discovered with molecular techniques. Initially, the role of such microorganisms for the tick or in the context of public health remains unknown. Several examples can be named here, ranging from probable endosymbionts of ticks like '*Candidatus *Midichloria mitochondrii' to '*Candidatus *Neoehrlichia mikurensis' which was found recently to be involved in several cases of severe human disease [1-9].

### *Wolbachia *in insects and its unclear role in ticks

Bacteria belonging to the group of *Wolbachia pipientis *have been detected in several studies in *Ixodes ricinus *ticks [10-13]. Bacteria from this group are known endosymbionts from a high variety of insects, mites and nematodes. They act, depending on the *Wolbachia *strain and host species, as mutualistic, commensalistic or parasitic symbionts [14-16]. *Wolbachia *have been shown to influence the reproduction of infected insects in various ways, including parthenogenesis, male killing, cytoplasmic incompatibility and feminization [16]. So far, the role of *Wolbachia *in ticks remained unclear [10,13]. Its prevalence was usually too low to be explained by obligatory symbiosis and it remained unclear how the ticks became infected. In the current study we offer an explanation for the source of *Wolbachia *sequences amplified from ticks.

### Ixodiphagus hookeri

Unexpectedly, three wasps hatched in our laboratory from an engorged nymphal *I. ricinus *tick. In accordance with the origin of the wasps we suspected these to be *Ixodiphagus hookeri *(Howard), a parasitoid of ticks belonging to the order Hymenoptera and the family Encyrtidae. *I. hookeri *is known to occur on all continents except for Antarctica and to infest various tick species [17]. So far, it had not been reported from the Netherlands.

Mating of *I. hookeri *occurs shortly after emerging from their tick host [15]. Females then search for a suitable tick host and oviposit several eggs per tick, for *I. ricinus *up to 15 wasps per nymph have been observed [18]. In a European setting unfed nymphs of *I. ricinus *are preferred above *I. ricinus *larvae and fed nymphs [18]. Worldwide at least 14 ixodid tick species, including several *Ixodes *spp., have been found to be suitable hosts. Suitability, however, might depend on the *I. hookeri *strain [17-19]. *I. hookeri *has been investigated for its potential as biological tick control agent with variable results [17,20].

### Study question

The discovery of parasitic wasps in Dutch ticks led to the suspicion that *Wolbachia *sequences from ticks previously identified in our laboratory and reported from other studies [10,12,13,21] might in fact be due to *Wolbachia *endosymbionts of tick parasitoids. We tested this by initially testing the parasitic wasp for *Wolbachia*. Ticks were examined for the presence of parasitoid DNA and *Wolbachia *by PCR to investigate associations between these two parameters. Additionally, the prevalence and distribution of *I. hookeri *in the Netherlands was investigated by testing ticks from various sites throughout the country.

## Methods

### *Ixodiphagus *source

Three specimens of parasitic wasp hatched from an engorged *I. ricinus *nymph that had been collected from sheep in a nature reserve area in the south-west of the Netherlands on September 7^th^, 2010. Ticks had been pooled per sheep and stored in 50 ml polypropylene tubes at room temperature, approximately 80% relative humidity and a natural day/night cycle. The parasitic wasps hatched between October 2010 and January 2011 and were discovered on January 10^th^, 2011. Two specimens were sent to the Natural History Museum, London, for taxonomic determination while the third specimen remained in our laboratory for molecular investigations. Total DNA was extracted from one specimen by alkaline lysis as described elsewhere [2].

### Ticks tested

Pilot test: From several studies from recent years (2008-2010) 27 *Wolbachia *positive ticks tested by reverse line blot (RLB) (as described in [12,22]) were selected. Additionally, one *Wolbachia *negative tick from the matching study was selected for each positive tick. In order to determine the distribution and prevalence of *I. hookeri *in the Netherlands adult and nymphal ticks from eleven different locations across the Netherlands were tested. Depending on availability, between 19 and 48 ticks per location were tested (for one location only 3 ticks were available). Total DNA was extracted from the ticks by alkaline lysis as described elsewhere [2].

### PCR *Ixodiphagus*

Several primer pairs (Table 1) targeting the 16S *rRNA *or 28S *rRNA *genes were tested on DNA extracted from an *I. hookeri *(Howard) specimen. As a positive control, DNA of *Vespula vulgaris *was used. PCR products were analyzed on agarose gels. All primer pairs yielded bands for *V. vulgaris *DNA and all but the 16S-F1/16S-R1 combination yielded PCR products for *I. hookeri (*Howard).

**Table 1:** Primers used for amplification and sequencing of *Ixodiphagus hookeri *(Howard) DNA

Primer	Target	sequence 5' → 3'	original name	Reference
16S-F1	16S rRNA	cacctgtttatcaaaaacat	16SWb	[32]
16S-F2	16S rRNA	ctgcagtattttgactgtacaaaggtagcataatc	--	This study
16S-R1	16S rRNA	cttaattcaacatcgaggtcgc	--	modified from [33]
28S-F1	28S rRNA	aagagagagttcaagagtacgtg	--	[34]
28S-F2	28S rRNA	actttcaggacccgtcttga	--	R/C of 28S-R1
28S-R1	28S rRNA	tcaagacgggtcctgaaagt	D2-4057 R	[35]
28S-R2	28S rRNA	tagttcaccatctttcgggtccc	28S-PM	[34]
28S-R3	28S rRNA	tcggaaggaaccagctacta	D3-4413 R	[35]

R/C: reverse-complement

Ticks from the pilot study were tested for the presence of Hymenoptera DNA by amplification of part of the 28S *rRNA *gene using primer pair 28S-F1/28S-R1 and part of the 16S *rRNA *gene using primer pair 16S-F2/16S-R1. Ticks from the prevalence study were tested with primer pair 28S-F1/28S-R1.

PCR was done using HotStarTaq master mix (Qiagen, Germany) with the following conditions: 15 min at 95°C, then cycles of 30 s at 94°C, 30 s at 65°C, 60 s at 72°C, lowering the annealing temperature by 1°C each cycle until reaching 55°C, then 35 cycles at this annealing temperature followed by a final elongation step for 7 min at 72°C. Samples were analyzed on agarose gels.

### PCR *Wolbachia*

DNA of the *I. hookeri *specimen and ticks were tested for the presence of *Wolbachia *DNA by amplification of part of the *wsp *gene using primer pair wsp81F/wsp691R (Table 2) [23]. PCR was done using HotStarTaq master mix (Qiagen, Germany) with the touch down PCR protocol as described above with a starting annealing temperature of 60°C and a final annealing temperature of 50°C.

**Table 2:** Primers used for amplification and sequencing of *Wolbachia *DNA

Primer	Target	sequence 5' → 3'	original name	Reference
wsp 81F	wsp gene	tggtccaataagtgatgaagaaac	wsp 81F	[23]
wsp 691R	wsp gene	aaaaattaaacgctactcca	wsp 691R	[23]
wspF2a	wsp gene	aaggccacagacattcataatccattaaaagcatc	--	This study
wspF2b	wsp gene	gcaacaggcaaagaaaaggatagtccct	--	This study
wspR2a	wsp gene	acagtgctgtaaaggactgtatgtcctcctttg	--	This study
wspR2b	wsp gene	acagtgctgtaaaggactgtatgtcctcctttg	--	This study

### Sequencing

PCR products were sequenced using an ABI PRISM Big-Dye Terminator Cycle sequencing Ready Reaction kit (Perkin Elmer, Applied Biosystems). All sequences were confirmed by sequencing both strands.

Sequencing of the *Wolbachia wsp *gene indicated double infections with *Wolbachia *in some tick samples and for these specimens additional sequencing primers were used (wspF2a, wspF2b, wspR2a, wspR2b) (Table 2).

Resulting sequences were compared with sequences in Genbank using BLAST.

### Statistical analysis

Positive and negative predictive values with regard to *Wolbachia wsp *PCR results as predictors for a positive and negative PCR results for Hymenoptera DNA were calculated. Additionally the significance of the association between these two PCRs was tested using Mid-P exact test [24].

## Results

### Identification of parasitic wasps

**Figure 1:**
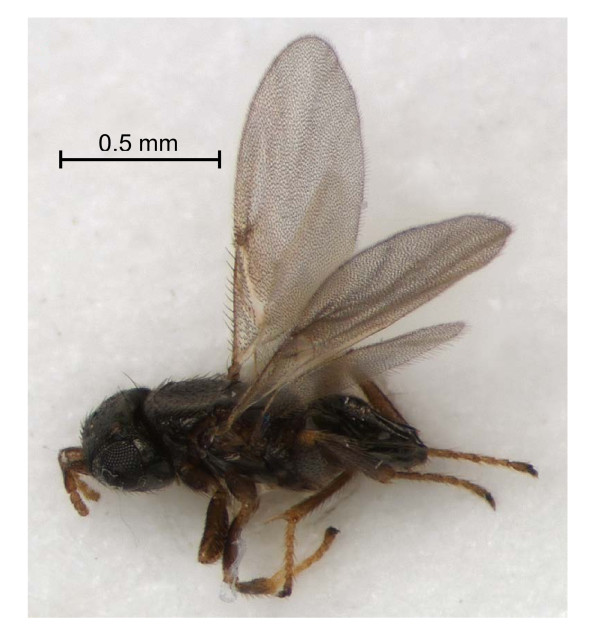
***Ixodiphagus hookeri *(Howard) hatched from an *Ixodes ricinus *nymph (Photo: Oscar Vorst, Naturalis)**.

The collected parasitic wasps were morphologically identified as *Ixodiphagus hookeri *(Howard) by John Noyes at the Natural History Museum, London (Figure 1). This species had previously not been reported from the Netherlands and has in the meantime been added to the list of Dutch Chalcidoidea and the Dutch Species Catalogue [25].

### PCR on *Ixodiphagus hookeri*

The 28S *rRNA *and 16S *rRNA *genes of *I. hookeri *were successfully amplified and sequenced. After clipping off the primer sites, sequences of 824 bp and 318 bp were obtained, respectively. BLASTing of the 28S *rRNA *and 16S *rRNA *sequences gave closest matches with the hymenopteran parasitoids *Plagiomerus diaspidis *(accession code AY599316, 84% identity) and *Cotesia flaviconchae *(AJ535921, 80% identity, 89% coverage), respectively.

**Figure 2:**
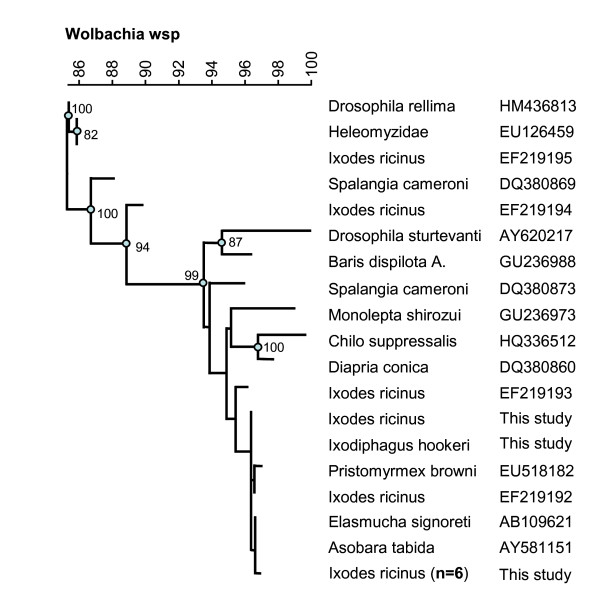
**Phylogenetic analysis of *Wolbachia *from various invertebrate host species**. Neighbor-joining trees with Kimura correction were based on *wsp *genes from this study and mined from GeneBank. Bootstrap proportions were calculated based on 500 replicates, only values < 80 are indicated.

Sequencing of the *Wolbachia wsp *gene yielded a sequence of 573 bp with no indications of double infection. BLASTing of the *wsp *sequence gave close matches with a number of *Wolbachia *endosymbionts of various insects. A phylogenetic tree based on the *wsp *gene was calculated (Figure 2).

### Pilot study

Fifty-four ticks were tested for the presence of *I. hookeri *and *Wolbachia *DNA. Amplification of the 16S *rRNA *gene yielded unspecific products, which were not used for further analysis. Amplified products of 28S *rRNA *and *wsp *genes were sequenced. Most RLB *Wolbachia*-positive ticks were positive for 28S *rRNA *as well as *wsp *genes. Five ticks of this group were positive for the *wsp *gene but delivered no or divergent 28S *rRNA *sequences. One tick was *I. hookeri *positive but did not produce a *wsp *amplification product. Four RLB *Wolbachia*-positive ticks were negative for either PCR. Twenty-four of 27 ticks from the RLB *Wolbachia*-negative control group yielded no PCR products for either 28S *rRNA *or *wsp *genes. Two ticks yielded 28S *rRNA *sequences different from *I. hookeri *and one tick yielded a *wsp *sequence different from the *I. hookeri *isolate. *Wolbachia wsp *sequences from several *I. hookeri *positive ticks failed to be sequenced in adequate length and quality, which was apparently due to double infection with multiple *Wolbachia *strains.

### Prevalence study

**Figure 3:**
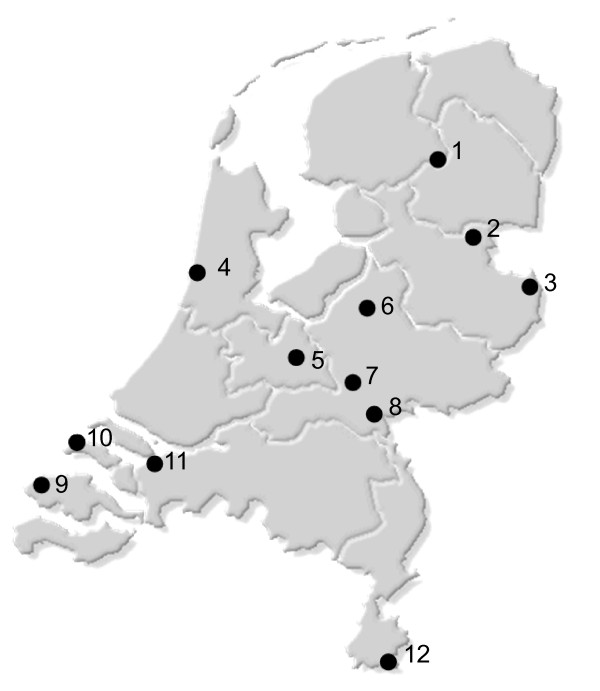
**Locations in the Netherlands with PCR verified presence of *Ixodiphagus hookeri *infested *Ixodes ricinus *ticks**. Drents-Friese Wold; 2. Boswachterij Hardenberg; 3. Landgoed Singraven; 4. Duin en Kruidberg; 5. Pyramide van Austerlitz; 6. Koninklijke Houtvesterijen Hoog Soeren; 7. Hullenberg; 8. Rijk van Nijmegen; 9. Kop van Schouwen; 10. Vrouwenpolder; 11. Dintelse Gorzen; 12. Vijlenerbos; Two of these sites (7 and 10) were found positive in the pilot study; the remaining sites were investigated in the prevalence study.

**Figure 4:**
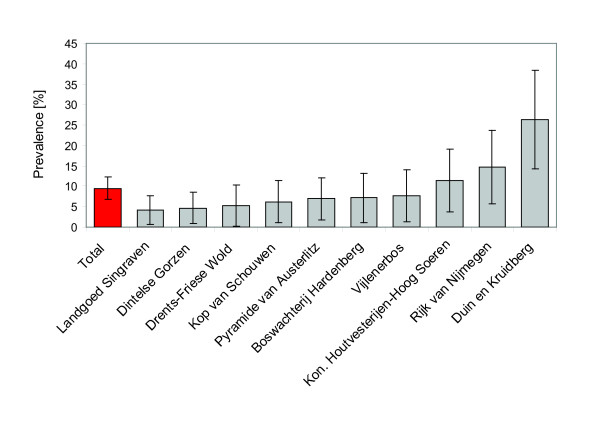
**Prevalence of the parasitic wasp *Ixodiphagus hookeri *in various locations spread over the Netherlands**. Error bars indicate 95% confidence intervals calculated with Mid-P exact test.

A total of 341 ticks were tested for the presence of *I. hookeri *PCR amplification of the 28S *rRNA *gene. Additionally 143 of these ticks were tested for *Wolbachia *by amplification of the *wsp *gene. *Ixodiphagus hookeri *DNA was detected in 9.7% (n = 33) of all ticks. Ten of eleven locations were found positive with a prevalence ranging from 4% to 26% (Figure 3 and 4). For the remaining site only 3 ticks were available, which were all negative. Of the 143 ticks tested for *Wolbachia*, 10.5% (n = 15) were found positive (Table 3). Two ticks that had been found positive for *I. hookeri *DNA were negative for *Wolbachia *and vice versa. A tick positive in the *Wolbachia *PCR had a probability of 87% to be positive for *I. hookeri *as well. Likewise, a negative *Wolbachia *result predicted a negative *I. hookeri *PCR with a probability of 98%. The association between PCR results for *Wolbachia *and *I. hookeri *DNA was statistically significant (p-value < 0.0001).

**Table 3:** Association of *Wolbachia wsp *and *I.hookeri *28S *rRNA *PCR results

		*Wolbachia *PCR		
		**positive**	**negative**	

*I. hookeri *PCR	positive	15	2	17
	
	negative	2	124	128

		17	128	143

## Discussion

DNA of *I. hookeri *was detected in ticks collected from locations broadly distributed across the Netherlands. Although this is the first notice of this species in the Netherlands the broad distribution and the cosmopolitan distribution of this insect indicates that the wasp is a native species. The prevalence varies strongly with sampling location as had been reported from other studies as well [26,27]. The prevalence might influenced by biological or climatic factors. Tick density, which in turn is affected by tick-host abundance, has been suspected to be a major determinant for the prevalence of *I. hookeri *[27]. This corresponds with the finding that the highest *I. hookeri *prevalence in this study was found in 'Duin & Kruidberg' (Figure 4), an area with exceptional high tick density (data not shown). However, another area with comparable tick density, 'Kop van Schouwe', had a much lower *I. hookeri *prevalence. Other factors like (micro)climate, vegetation and prevalence of different vertebrate species in the habitats might play a role, but such data were not collected during this study.

The molecular data suggest that *I. hookeri*, as many other insects, may harbour at least one *Wolbachia *strain. The specimen studied here was infected with a *Wolbachia *strain similar to that of other Hymenoptera but also closely matched *Wolbachia *endosymbionts of insects from different orders. Although the specimen hatched in our laboratory was apparently infected with a single strain, several *I. hookeri *positive ticks harboured multiple *Wolbachia *strains. Multiple infections with *Wolbachia *are known to occur in insects [28,29] and the combination of these might have different biological impacts on their hosts. *Wolbachia *have been linked to parasitoid specialization [30] and different *Wolbachia *strains in *I. hookeri *might be one explanation for apparent differences in its biology in different countries [18,19,31].

Detection of *Wolbachia *DNA in ticks in this study is strongly correlated with *I. hookeri *infestation. The small deviation between PCR results might be explained by different sensitivities of PCRs. On the one hand, some *I. hookeri *might have no or only a low *Wolbachia *load while others might have a high bacterial load. On the other hand, the copy number of the 28S *rRNA *gene, which was used to detect *I. hookeri *in this study, is not known for this species and will influence the PCR sensitivity for this target. Furthermore PCR products from the prevalence study were not sequenced. Therefore, some of the *wsp *and 28S *rRNA *positive PCR results might have been due to environmental contamination of samples with *Wolbachia *or insect species not related to infestation of ticks.

Data on spread and prevalence of *I. hookeri *around the world is scarce. The specificity of the *I. hookeri *PCR could be increased for future studies by the use of primers with high specificity, which can now be designed based on the 16S *rRNA *and 28S *rRNA *sequence data from this study. In future, molecular methods can be used to examine the spread and prevalence of *I. hookeri *in different tick species and regions.

## Conclusions

We report for the first time the detection of *I. hookeri *in Dutch ticks and the presence of *Wolbachia *in *I. hookeri*. This is, to our knowledge, the first reported study investigating the prevalence of *I. hookeri *in tick populations with molecular methods. *I. hookeri *seems to harbour *Wolbachia *endosymbionts, which can explain previous reports of *Wolbachia *isolates from *I. ricinus *ticks. In case they are endosymbionts of parasitoids, and not of the ticks themselves, these *Wolbachia *are unlikely to play a role in human diseases.

## Competing interests

The authors declare that they have no competing interests.

## Authors' contributions

ETK performed laboratory analysis, analyzed data and wrote the first draft. MB coordinated wasp identification and contributed to the study design. EJS collected and provided ticks. HS contributed to the study design and wrote the final manuscript. All authors approved the final version of the manuscript.
